# Coral-like silicone nanofilament coatings with extremely low ice adhesion

**DOI:** 10.1038/s41598-021-98215-1

**Published:** 2021-10-14

**Authors:** Davide Bottone, Valentina Donadei, Henna Niemelä, Heli Koivuluoto, Stefan Seeger

**Affiliations:** 1 Department of Chemistry, University of Zurich, Winterthurerstrasse 190, 8057 Zurich, Switzerland; 2 Materials Science and Environmental Engineering, Faculty of Engineering and Natural Sciences, Tampere University, P.O. Box 589, 33014 Tampere, Finland

**Keywords:** Nanoscale materials, Design, synthesis and processing, Wetting

## Abstract

Passive icephobic surfaces can provide a cost and energy efficient solution to many icing problems that are currently handled with expensive active strategies. Water-repellent surface treatments are promising candidates for this goal, but commonly studied systems, such as superhydrophobic surfaces and Slippery Liquid Infused Porous Surfaces (SLIPS), still face challenges in the stability and durability of their properties in icing environments. In this work, environmental icing conditions are simulated using an Icing Wind Tunnel, and ice adhesion is evaluated with a Centrifugal Adhesion Test. We show that superhydrophobic coral-like Silicone Nanofilament (SNF) coatings exhibit extremely low ice adhesion, to the point of spontaneous ice detachment, and good durability against successive icing cycles. Moreover, SNFs-based SLIPS show stably low ice adhesion for the whole duration of the icing test. Stability of surface properties in a cold environment is further investigated with water wettability at sub-zero surface temperature, highlighting the effect of surface chemistry on superhydrophobicity under icing conditions.

## Introduction

Every year, icing affects several sectors of human activity, such as air^1,2^ and maritime^3^ transport, off-shore operations^3^, and power generation^4^ and delivery^5^. Addressing the resultant loss in efficiency or outright interruption of function is costly and energy intensive, and often involves significant safety risks. For this reason, the development of passive icephobic surface treatment, i.e. treatments that can reduce ice accumulation and decrease its adhesion to the substrate without external stimuli other than gravity, wind or surface tension^6^, has been the subject of extensive research^7–9^. However, how ice is accreted on a surface influences significantly its ice adhesion^10^, which in turn is strongly dependent on the mode of ice removal^11–13^, and considerable challenges still exist on the standardization of ice adhesion testing^8,12,13^.

Since the search for an ideal icephobic surface began in the late 1930s, it’s been debated whether water wettability plays a role on ice adhesion^14,15^. Initial reports stated that a hydrophobic surface chemistry is beneficial against ice adhesion^14^, although it should be appropriately coupled with other material parameters, such as roughness and mechanical properties^15^. Moreover, the existence of a quasi-liquid layer on the ice surface, which confers its molecularly slippery character^16^, suggests that water mobility on the material surface might play a direct role on ice adhesion^15,17^. Ultimately, it has been demonstrated that ice adhesion correlates well with the practical work of adhesion ${W}_{\text{a}}=\gamma \left(1+{\mathrm{cos}\theta }_{\text{rec}}\right)$, where *γ* is the surface tension of water, and ${\theta }_{\text{rec}}$ is the receding water contact angle^18^. However, this correlation is strictly valid only for nominally smooth surfaces and relatively high ice adhesion strength (> 160 kPa)^19,20^, and a more intricate interplay of material properties is at the basis of the extremely low ice adhesion of more complex systems.

Superhydrophobic surfaces (SHS), that owe their extreme water repellency to the *Cassie-Baxter* wetting state of the biomimetic *Lotus effect*^21^, are seen as promising anti-icing systems^22–25^. The air cushion trapped within their surface texture contributes to reducing ice accumulation by delaying freezing of impacting water droplets thanks to its low heat transfer coefficient^26–28^, and, once ice has started to accrete, facilitates its removal by promoting interfacial crack initiation^29,30^. However, many SHS are extremely sensitive to environmental conditions, such as pressure and humidity, and failed to show icephobic behavior, with the exact surface texture geometry being critical for ice adhesion^11,30–32^.

To overcome these shortcomings, slippery liquid infused porous surfaces (SLIPS) have been tested in anti-icing applications^33–36^. The presence of a stabilized liquid lubricant layer, inspired by the slipperiness of the *Nepenthes* pitcher plant^37,38^, grants SLIPS a water repellency that is much more stable to pressure and humidity compared to SHS, as well as a very mobile ice-substrate interface with extremely low ice adhesion strength. However, long term stability of these surfaces towards lubricant depletion under flow and frost exposure remains a significant challenge^39,40^.

A key issue in transferring any material from fundamental research to a potential application is ensuring its ability to maintain the required function under continuous operation. Icing is no exception to this statement: a passive icephobic surface will be subjected to multiple ice accretion/ice detachment cycles during the course of a single icing event. Therefore, it’s necessary to prove not only that the ice adhesion strength to a given surface is low enough for the specific application, but also that its icephobic performance doesn’t significantly degrade over consecutive icing cycles.

In the present work, superhydrophobic hierarchical coral-like Silicone Nanofilaments (SNFs) were synthesized on anodized aluminum through the vapor-phase Droplet Assisted Growth and Shaping (DAGS) route^41–43^. This process allows the growth of different polysilsesquioxane micro- and nanostructures with controllable shape on a variety of different substrates^42^, and is well suited for industrial upscaling^44^. The obtained coatings show excellent chemical durability^41,42^, can be easily subjected to further functionalization, and are resistant to outdoor weathering and UV exposition^45^. Moreover, the small scale porosity of the coatings makes them suitable to fabricate Slippery Liquid Infused Porous Surfaces (SLIPS) by infusing them with lubricants^46^.

Impacted ice was accreted from supercooled water droplets on top of the coral-like SNFs and SNFs-based SLIPS by using an icing wind tunnel (IWiT) to simulate environmental icing conditions. Ice adhesion strength on the surfaces was then evaluated with a Centrifugal Adhesion Test (CAT) throughout 5 consecutive ice accretion—ice detachment cycles, while also monitoring the evolution of their wetting behavior and, for the non-infused surfaces, their microstructure and surface chemistry. Moreover, wettability of the surfaces at low temperatures (− 10 °C) was investigated, and its relationship with ice adhesion was examined.

## Results and discussion

Tested surfaces and their respective sample names are summarized in Table 1.

**Table 1:** Sample names and description.

Sample name	Sample description
S	Coral-like silicone nanofilaments (SNFs)
F	Fluorinated SNFs (f-SNFs)
LS	SNFs infused with silicone oil
LF	SNFs infused with perfluorinated oil

### Coral-like silicone nanofilaments

**Figure 1:**
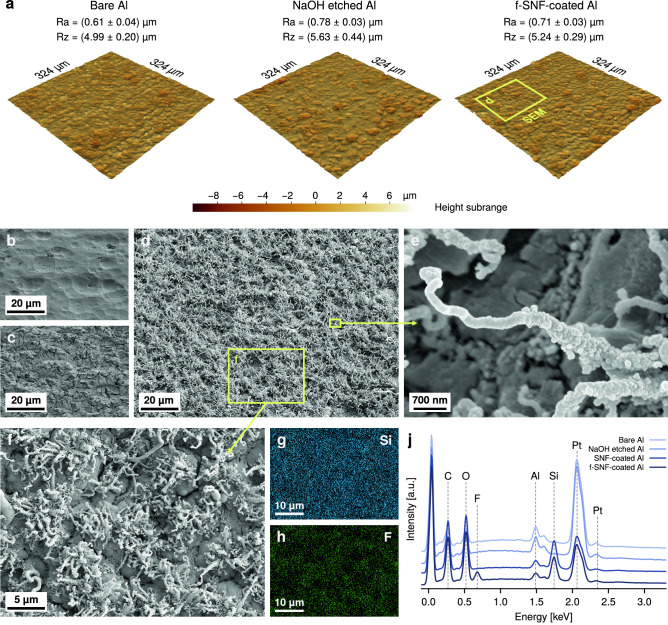
Surface micro- and nanostructure of SNF-coated Al samples. (**a**) Optical surface roughness maps of bare Al substrate, NaOH etched Al substrate and fluorinated-SNF-coated Al (F). (**b**–**e)** SEM micrographs of: **b** bare Al substrate, (**c**) NaOH etched Al substrate, and (**d**, **e)** sample F. (**f**) SEM micrograph of sample F, with its EDX maps of (**g**) Si and (**h**) F. (**j**) EDX spectra of bare Al substrate, NaOH etched Al substrate and SNF-coated Al before and after fluorination.

Figure 1 shows the SNF-coated Al samples’ surface topography at different scales and at different stages of the fabrication process. In Fig. 1a surface maps obtained via optical profilometry are displayed together with the measured roughness values of Ra and Rz, showcasing the surface microstructure of the samples over the course of the fabrication steps. It is apparent that, aside from shallow pits introduced during the etching step, clearly shown in Figs. 1c and S1a,b, no major change in surface topography is appreciable at the observed length scale. Moreover, it appears that the coating process does not significantly alter profile roughness as measured by this technique. Optical profilometry is however not able to effectively resolve the sub-micrometric roughness introduced by the DAGS process: on the other hand, SEM images of the SNF- and fluorinated SNF-(f-SNFs) coated Al, respectively shown in Fig. S1c,d and Fig. 1d,e, clearly reveal the presence of a layer of coral-like SNFs. These nanostructures are intrinsically hierarchical, with several stalks developing from one common point and possessing smaller protuberances that hint to a side growth from the main structure. Moreover, the fluorination step does not influence SNF morphology.

Comparison of the EDX spectra of the samples before and after SNF coating (Fig. 1j) confirms the introduction of a polysilsesquixane layer, as a strong Si signal is only present on the coated samples; while also C and O are present in the polysilsesquioxane layer, the change in surface morphology arising from the coating precludes a direct comparison with the pre-existing signals in the uncoated samples. Additionally, the F peak observed on the f-SNF-coated sample confirms the successful fluorination step.

EDX element mapping of the coated samples further supports the presence of a homogeneous polysilesquioxane coating (Fig. S1e,f) that is retained after fluorination, as shown in Fig. 1f–g. The fact that the Si signal does not appear to be specifically associated with the coral-like stalks of the SNFs points out the presence of a polysilsesquioxane underlayer below the SNFs, as already observed for other DAGS-derived coating^47^. Moreover, EDX mapping of F on f-SNFs, shown in Fig. 1h, confirms the spatial homogeneity of the fluorination.

Owing to the hierarchical structure and hydrophobic surface chemistry of the coral-like SNFs, the coated samples, both fluorinated (F) and non-fluorinated (S), are perfectly superhydrophobic, showing water contact angles greater than 170° and sliding angles lower than 2°. Moreover, when infused with a lubricating oil, coral-like SNFs are able to retain the lubricant and form an efficient SLIPS. The obtained slippery surfaces display water sliding angles lower than 3° for both silicone oil (LS) and perfluorinated oil (LF) infusion.

### Ice adhesion testing

There is no consensus on a general threshold value of ice adhesion strength for extremely icephobic materials: this stems from the fact that the measured ice adhesion is highly dependent on the specific testing conditions, such as ice type, accretion method and the ice adhesion test itself^48^. For this reason, in general, no direct comparison between results obtained with different testing techniques can be carried out without previous calibration^12^. Therefore, such threshold necessarily needs to be defined for each specific testing setup based on its experimental history. For the Tampere University’s icing facilities, a first threshold for low ice adhesion materials was defined in previous work at an adhesion strength value of 50 kPa, while extremely icephobic materials were defined as those possessing ice adhesion strength values below 10 kPa^36,48^.

**Figure 2:**
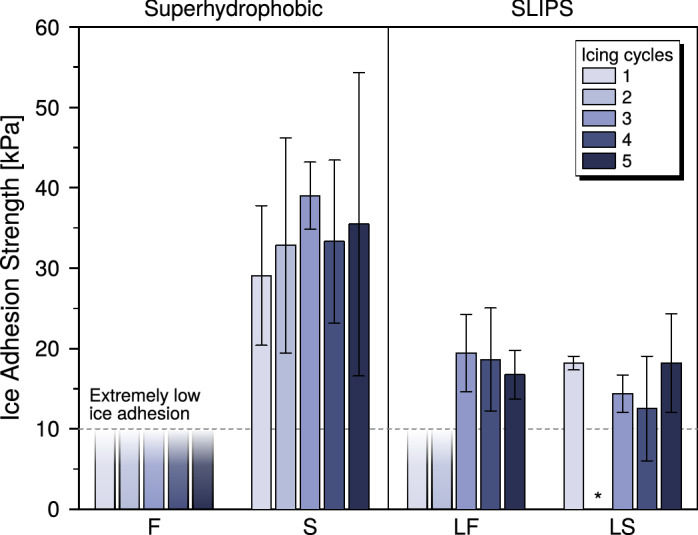
Ice adhesion results for all samples and icing cycles. Error bars are equal to one standard deviation. It was not possible to definitively assign a value to sample LS at its 2nd icing cycle (marked with an asterisk*).

Results of the ice adhesion testing are shown in Fig. 2, where it can be clearly seen that all tested coatings have an ice adhesion strength lower than 50 kPa, and therefore they all can be considered as low ice adhesion surfaces. Sample S showed an ice adhesion strength (29 kPa ± 8 kPa on the pristine sample) slightly lower, but comparable, to that of superhydrophobic surfaces previously measured with the same setup (43 kPa)^49^ and of nanostructured silicone rubber measured in similar conditions (38 kPa)^50^. In addition to that, the values measured for SLIPS (LS and LF) lay between 10 and 20 kPa, and fall within the range observed for slippery and lubricated surfaces tested using the same IWiT and testing method^35,36,48,51^. Notably, although a direct comparison is not possible, LS and LF show values close to those observed for SLIPS with other characterization methods relative to reference surfaces^33,34,47^.

Moreover, when testing sample F, ice detached spontaneously during handling of all the iced specimens while transitioning from the ice accretion to the ice adhesion testing phase; Supporting Fig. S1 shows an example of the cleanly sheared ice blocks observed. This behavior is clearly revealing of the extremely icephobic character of the tested surface, which, however, lies below the sensitivity of the employed characterization technique. It is therefore our assumption that the actual ice adhesion strength of sample F is lower than the threshold value of 10 kPa reported for extremely icephobic surfaces, although we have no elements to precisely assign a numerical value to it. Moreover, this detachment under very low external loads is qualitatively indicative of the good ice shedding behavior the coating might show in a real-world application^8^. To the best of our knowledge, such behavior has not been previously reported for superhydrophobic surfaces with ice accreted in similar conditions. A similar behavior was also observed for all specimens of sample LF tested in the first and second icing cycle, but a finite value of ice adhesion strength was measured starting from the third cycle, implying a decrease in the surface icephobicity.

It is also evident from Fig. 2 that no significant degradation in ice adhesion properties can be observed with increasing number of icing cycles, with the exception of sample LF. It should be noted that, for the aforementioned reasons, it is not possible to detect a subtle change in the ice adhesion strength of sample F; however, it is apparent that the sample keeps its extreme icephobicity throughout the entire testing procedure. This is especially remarkable because of the high relative humidity of the accretion environment (approximately 80%^51^), which has been reported to cause critical failure of the anti-icing behavior of superhydrophobic surfaces^28,30,52,53^.

A recent work also investigated ice adhesion on superhydrophobic and liquid-infused SNFs^47^: while, similarly to the present work, SNFs were out-performed by f-SNFs and liquid-infused SNFs, the dramatic spontaneous ice detachment observed here for coral-like f-SNFs did not occur. A number of reasons could explain this different behavior. Both works studied DAGS-derived coating, but the actual morphology of the nanostructures differed greatly, with the notable feature that the coral-like SNFs shown here possess intrinsically multi-scale hierarchical features, which should improve impalement resistance against impacting droplets and favor the stability of a superhydrophobic state, as will be discussed later^54^. Moreover, the two studies employed substrates with different mechanical properties, and, most of all, used different ice adhesion testing protocols. The combination of these facts makes so that the results cannot be directly compared, and ongoing effort is directed at bridging this gap. This further highlights the need for inter-laboratory cooperation to develop standardized ways of comparing different ice adhesion tests.

**Figure 3:**
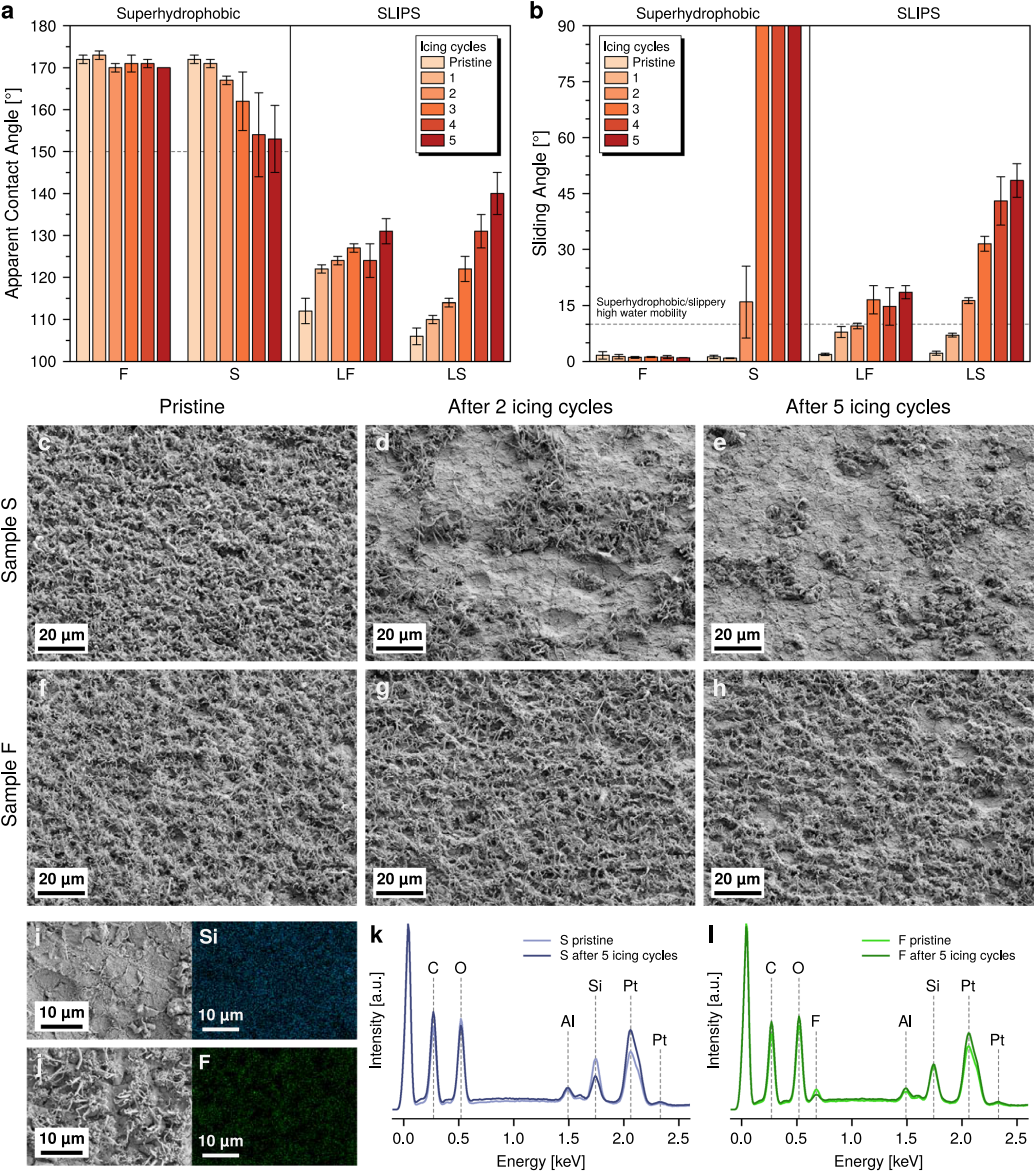
Durability of surfaces against ice adhesion. (**a**) Apparent water contact angle and (**b**) water sliding angle measured on samples before adhesion testing and after each icing cycle. The dashed line in (**a**) represents the lower contact angle threshold for superhydrophobic behavior. (**c**–**h)** Low magnification SEM micrographs showing the evolution of the SNFs coating on (**c**–**e)** sample S and (**f**–**h**) sample F with increasing number of icing cycles. (**i**) SEM micrograph of sample S after 5 icing cycles (left) and its EDX Si map (right). (**j**) SEM micrograph of sample F after 5 icing cycles (left) and its EDX F map (right). (**k**, **l**) EDX spectra of (**k**) sample S and (**l**) sample F before and after ice adhesion testing.

Further understanding of the different mechanisms at the basis of the surfaces ice adhesion behavior can be gained from their durability, by observing how ice accretion and ice removal affected their properties. Figure 3 shows the surfaces durability against icing cycles in the adhesion testing, in terms of degradation of wetting properties and changes to surface morphology and chemical composition.

From Fig. 3b it can be seen that a significant increase in water sliding angle was measured on the slippery surfaces LF and LS with successive icing cycles, pointing out at an ongoing lubricant depletion process^46^. A corresponding increase in apparent water contact angle was observed for the same surfaces, as shown in Fig. 3a. A clarification is necessary on the interpretation of apparent contact angle measurements on SLIPS: the method here employed, which consists in the extrapolation of the droplet profile away from its contact point with the surface, does not directly take into account the presence of an annular wetting ridge of lubricant around the droplet base^55,56^. Nevertheless, while this approach does not give a full picture of the complex interplay of the interfacial tensions between the four phases present, it can still yield meaningful information on the filling state of the nanostructure, with an increase in apparent contact angle being indicative of lubricant depletion^46^. Indeed, this is due to the reduction in the wetting ridge height accompanying a lubricant layer height reduction^56^, which in turn causes the sessile water droplet to assume a more spherical shape.

Comparing the trends of the increase in sliding angle and apparent contact angle of surfaces LF and LS, it appears that sample LS underwent a faster depletion than sample LF. In the tested icing conditions, lubricant is primarily removed from the infused surface by capillary wicking into the accreted ice porosity^40,57^. Assuming as a first approximation that the infused lubricant acts as an infinite liquid reservoir and that the capillary rise in the accreted ice follows Washburn’s law^58^, the height *z* reached in a given time *t* is given by Eq. (1):

1 $$z \sim {\left(\frac{\gamma }{\eta }t\right)}^\frac{1}{2}$$

where *γ* is the surface tension and *η* is the dynamic viscosity of the liquid. As shown in Table 2, the perfluorinated oil of sample LF had a much higher kinematic viscosity at room temperature than the silicone oil of LS. Moreover, this difference is heightened at the lower temperatures of the ice adhesion test, resulting in the perfluorinated oil having a kinematic viscosity approximately 30 times larger than that of silicone oil; taking also into account its higher density, this leads to an even larger difference in terms of dynamic viscosity. From Eq. (1), the much higher dynamic viscosity results into a lower lubricant capillary rise for sample LF, since all surfaces are in contact with ice for the same time. The lower surface tension of the perfluorinated oil can also contribute in the same direction, but in this case the difference with silicone oil is much less pronounced, as also shown in Table 2.

**Table 2:** Physical properties of lubricant oils at room temperature (25 °C) and − 10 °C.

Lubricant oil	Kinematic viscosity *ν*^a^ (mm^2^ s^−1^)		Surface tension *γ *(mN m^−1^)		Density *ρ *(g cm^−1^)
	25 °C	− 10 °C	25 °C	− 10 °C	25 °C
Perfluorinated oil	390 ± 14	6651 ± 815	19^36^	22^59^	1.9^b^
Silicone oil	100 ± 1	219 ± 8	21^36^	23^60^	0.96^b^

^a^Kinematic viscosity temperature dependence determined in accordance with ASTM D314-17.

^b^Data from manufacturer.

While the surfaces are exposed to the supercooled droplet-carrying IWiT airflow, which can cause lubricant depletion by interfacial shear forces^39^, they are very quickly covered by an initial layer of accreted ice, reducing the contribution of this mechanism to the overall lubricant depletion.

Figure 3 also shows the variation of contact angle (Fig. 3a) and sliding angle (Fig. 3b) of the superhydrophobic F and S surfaces. It can be clearly seen that the fluorinated coral-like SNFs of sample F maintained their superhydrophobic behavior for all the duration of the test, without any observable degradation in wetting properties. On the other hand, the non-fluorinated SNFs of sample S started to lose their superhydrophobicity after the second icing cycle, with a complete pinning of water droplets by the third icing cycle. Compared to SLIPS, superhydrophobic surfaces undergo a fundamentally different type of degradation when subjected to repeated ice accretion/ice detachment cycles. Indeed, the comparatively high aspect ratio of the nano- or micro-structures necessary to obtain superhydrophobic behavior often easily expose them to mechanical damage when accreted ice is removed^8,52^. This is evident from Fig. 3c–e, where a progressive removal of patches of SNFs from the surface of sample S is clearly visible, which in turn results in the loss of its superhydrophobic character. On the other hand, Fig. 3f–g shows that little to no damage to the fluorinated SNFs can be observed with successive icing cycles. Moreover, higher magnification SEM images of sample F (Fig. S3c,d) show that the nanostructure is fundamentally unchanged by the ice adhesion testing procedure, while in sample S (Fig. S3a,b) the longer SNFs are often collapsed or cut off, when not removed entirely.

However, the homogeneous distribution of Si observed from the EDX map of sample S, shown in Fig. 3i, points out that the polysilsesquioxane underlayer beneath the SNFs withstands the aggressive cycling, as observed for DAGS-derived coatings on other substrates^47^. On the other hand, the F EDX map of sample F after ice adhesion testing (Fig. 3j) confirms that a fairly homogenous fluorination is retained after ice adhesion testing. Furthermore, EDX spectra of samples S and F (Fig. 3k,l) show that no significant change in surface chemistry is underwent by the samples as a result of the icing test.

The stark difference in durability observed between samples S and F can be explained by the different state of the accreted ice on the two surfaces. When ice is accreted on a superhydrophobic surface, it can either rest on the surface texture asperities, keeping the air pockets characteristic of superhydrophobic surfaces, or displace the trapped air layer and form inside the surface texture, resulting in a high degree of mechanical interlocking^30^. These two states are usually referred to, respectively, as *Cassie* ice and *Wenzel* ice, by analogy with the wetting states of a sessile droplet on a textured surface. The existence of a *Cassie* ice state is necessary to ensure extremely low ice adhesion on superhydrophobic surfaces^29,30^. The damage observed to the sample S nanostructure, together with its comparatively higher ice adhesion strength, supports the hypothesis that the accreted ice was in a *Wenzel* state, and it sheared off the interlocked SNFs when removed from the surface. On the other hand, the lack of substantial microstructural damage and the extremely low ice adhesion observed on sample F confirms the existence of a *Cassie* ice state on the surface. This result also has the remarkable side effect that the surface with the lowest ice adhesion also shows the best durability against icing. Finally, it should be noted that the flexibility of SNFs may contribute to interfacial mobility and to decrease ice adhesion strength, although further investigation on the effect of soft nano- and micro-structures is required.

However, the virtually identical superhydrophobic character of the pristine S and F samples do not explain the sharp difference observed in their anti-icing behavior, and further analyses were necessary to elucidate it.

### Role of wetting on ice adhesion

Sessile droplet measurements are usually conducted at room temperature. However, temperature can dramatically alter the delicate equilibrium of interfacial tensions that dictates a sessile droplet contact angle and its hysteresis, with significant repercussions on water mobility on the surface.

**Figure 4:**
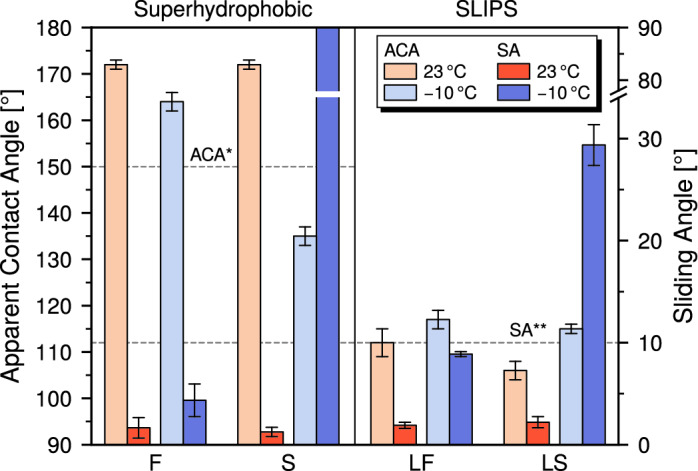
Apparent water contact angle (ACA) and water sliding angle (SA) measured on pristine samples at surface temperature 23 °C and − 10 °C. Error bars are equal to one standard deviation. ACA*: lower CA threshold for superhydrophobic behavior; SA**: upper SA threshold for superhydrophobic and slippery behavior.

Figure 4 shows the results of contact angle and sliding angle measurements on the tested SNF-coated materials at different surface temperatures. It can be clearly observed that, while sample F retains its superhydrophobic character at − 10 °C, sample S shows a significant decrease in contact angle and complete pinning of water droplets on its surface. This is evidence of a temperature-dependent *Cassie* to *Wenzel* transition on non-fluorinated SNFs, and well matches the observed ice adhesion strength and microstructural durability results, which in turn support a *Wenzel* ice state on sample S. Such a transition can be expressed in terms of the *Young* contact angle ${\theta }_{\text{E}}$^61^, that is the water contact angle on an ideally flat surface with the same surface chemistry of the real surface considered, as in Eq. (2):

2 $$\mathrm{cos}{\theta }_{\text{E}} > \frac{\phi - 1}{\phi - r}$$

where *ϕ* is the solid fraction in contact with liquid and *r* is the ratio between the real and the apparent surface area. That is, if the *Young* contact angle drops below a certain threshold given by the surface geometry, a *Cassie* to *Wenzel* transition occurs. Moreover, a significant contribution to the reduction of ${\theta }_{\text{E}}$ is given by water condensation (and possibly frosting) on the surface, which can render the surface chemistry substantially more hydrophilic and is indeed the main reason for the failure of superhydrophobic surfaces in anti-icing applications^30^. It is therefore clear that, for the specific texture geometry given by coral-like SNFs, a perfluorinated surface chemistry is necessary to maintain superhydrophobicity at low temperatures. This is consistent with previous work highlighting the effect of surface chemistry on superhydrophobicity at low temperature^62–64^. Surfaces showing low sliding angle and contact angle hysteresis at low temperature were also found to possess the lowest ice adhesion strength^62–64^, although, to the best of our knowledge, never before against impact ice.

**Figure 5:**
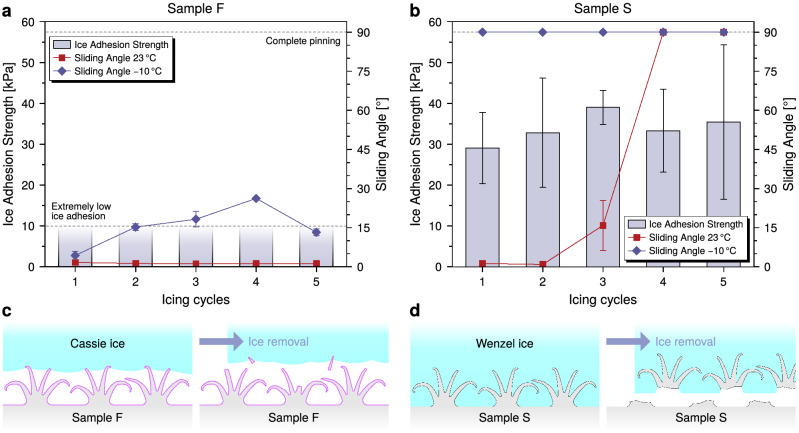
Ice adhesion and wetting at different temperatures. (**a**, **b**) Correlation between ice adhesion and wetting at 23 °C and − 10 °C for (**a**) sample F and (**b**) sample S. The values of sliding angle reported at a given cycle are those measured before the cycle. Error bars are equal to one standard deviation. (**c**, **d**) Schemes of Cassie ice on sample F (**c**) and Wenzel ice on sample S (**d**) and their respective mode of damage to the surfaces.

Ice adhesion strength and water sliding angle at 23 °C and − 10 °C are plotted together for successive icing cycles in Fig. 5. It’s evident that sample F (Fig. 5a), while not strictly superhydrophobic at − 10 °C after a few icing cycles, maintains a certain degree of droplet mobility on its surface, supporting the existence of a *Cassie* ice state for the whole duration of the test (Fig. 5c), as highlighted by the extremely low ice adhesion. On sample S (Fig. 5b), no clear transition in ice adhesion strength accompanies the complete loss of superhydrophobicy at 23 °C shown by the sample after the 3rd icing cycle. On the other hand, the stable *Wenzel* water wetting state at − 10 °C well matches the nearly constant higher ice adhesion strength of the material, which, together with the microstructural damage shown in Fig. 3c–e, can be indicative of a *Wenzel* ice state (Fig. 5d). It is therefore clear that a superhydrophobic behavior at room temperature is not a guarantee of superhydrophobicity at freezing conditions, nor, most of all, of low ice adhesion^11,30,31^. However, it is our belief that the more demanding characterization of wettability at low temperature and, possibly, at high relative humidity might be an appropriate screening tool for icephobic coatings, and its correlation with ice adhesion strength should be systematically investigated for superhydrophobic and lubricant infused surfaces. Particularly, this approach could allow for effective surface texture optimization of fluorine-free superhydrophobic coatings to maintain their properties at low temperatures, before validation in the more costly and time-consuming icing experiments.

A significant increase in sliding angle, coupled with a less pronounced increase in apparent contact angle, was also observed on pristine SLIPS tested at different surface temperatures, as shown in Fig. 4. Nevertheless, while hindered, water droplets always remained mobile on the surfaces. This behavior is revealing of contact line pinning, which can only occur on the asperities of the solid texture. However, differently from Fig. 3b, it cannot be ascribed to lubricant depletion, as the analysis was conducted on pristine surfaces. Instead, it could arise from a different thermodynamic state of the liquid infused surface^65,66^. Change in interfacial energies due to the temperature decrease may cause a transition in the lubrication state of the micro-nanostructure. It’s possible that, as a result of this change, the lubricant may be partly or fully displaced from the micro-nanostructure by the water droplet, causing the exposure of surface asperities to water and the creation of potential pinning points. Emergent surface asperities have actually been identified as a possible crack initiation points of the surface-accreted ice interface^34^, which could explain why no clear increase in ice adhesion strength can be observed on sample LS as depletion progresses. The increase in sliding angle is significantly larger on the silicone oil-infused LS compared to the fluorinated oil-infused LF, which further supports the origin of the phenomenon laying in the change of interfacial energies and lubrication status. Moreover, this difference could explain why ice adhesion on pristine sample LS is significantly higher than that on pristine sample LF. Further work is however required to fully elucidate the effect of low temperature on the lubrication status of SLIPS and explore its possible correlation with ice adhesion, and particular effort should be directed towards a rigorous verification of stability criteria at different temperatures.

A final point should be raised on droplet impact. When micrometric-sized droplets, such as those commonly present during icing events, impact on textured surfaces of comparable size, the geometry of impact can lay outside the range of validity of current models, and growing evidence supports the existence of non-wetting states when wetting would instead be predicted^32^. Moreover, material elasticity also plays a role in extending the range of stability of a non-wetting state^67^, and flexible textures such as SNFs might contribute to this effect, both by restituting kinetic energy to the impacting droplet and allowing for local increases of capillary pressure. Future work should therefore aim to the systematic extension of droplet impact models to complex surface textures and micrometric droplets, as well as study the possible role of soft and flexible textures, in order to have reliable predictive models for material behavior in impact ice conditions.

## Conclusions

One of the main sources of the icephobic character of superhydrophobic surfaces and SLIPS is the low wetting and the high mobility of water that comes in contact with them. Thanks to these properties, impacting water droplets can be shed away, thereby retarding ice accumulation, and the accreted ice can be easily removed owing to a combination of favorable interface shape, high interfacial mobility, and low chemical affinity to the surface. However, especially for superhydrophobic surfaces, maintaining these properties at the low temperatures and high relative humidities typical of environmental icing events is not trivial, and requires accurate design of the surface chemistry and morphology.

Hierarchical coral-like SNF coatings grown of anodized Al substrates through the DAGS process were shown to be effective as low adhesion surfaces against impact ice, both as intrinsically superhydrophobic surfaces and when infused with lubricant oil as SLIPS, with no significant degradation of icephobic character with successive ice adhesion/ice detachment cycles. Surface chemistry was observed to play a significant role in the ice adhesion of superhydrophobic surfaces, with non-fluorinated surfaces displaying considerably higher ice adhesion strength and substantial microstructural damage as testing progressed, while fluorinated surfaces showed extremely low ice adhesion below the sensitivity of the characterization technique, consistently outperforming the tested SLIPS.

Wetting characterization at low temperature, intended to mimic the environmental conditions found during the icing test, revealed that non-fluorinated coral-like SNFs failed to maintain their room-temperature superhydrophobicity, leading to a *Wenzel* ice state and higher ice adhesion, while fluorinated SNFs and SLIPS showed a high degree of water mobility even in these conditions. It is therefore suggested that wetting characterization at low temperature and, possibly, high relative humidity might be a more appropriate tool for the screening of prospective icephobic coating based on water repellency compared to its room temperature counterpart, although ice adhesion testing is ultimately required to assess the icephobicity of a surface.

Future work should focus on the long-term durability of fluorinated coral-like SNFs coatings and coral-like SNF-based SLIPS in large scale real-world environmental icing tests, to definitively assess the potential of these promising surfaces as passive anti-icing solutions. Moreover, further research should be directed toward the extension of current models to the study of supercooled micrometric droplet impacts on textured surfaces, in order to create a robust predictive framework to guide the rational design and optimization of these systems.

## Methods

### Materials

Trichloro(methyl)silane (TCMS; 98%; CAS: 75-79-6), Trimethoxy(methyl)silane (TMMS; 98%; CAS: 1185-55-3), Trichloro(1H,1H,2H,2H-perfluoro-1-octyl)silane (TCPFOS; 97%; CAS: 51851-37-7), and Silicone oil (viscosity 100 cSt at 25 °C, CAS: 63148-62-9) were acquired from Sigma-Aldrich (US). Sodium Hydroxide (NaOH; ACS/ISO compliant, CAS: 1310-73-2) was acquired from Merck Millipore (DE). Krytox GPL105 (viscosity 522 cSt at 20 °C) was acquired from DuPont (US). Anodized aluminum sheets (EN AW-5005/EN AW-AlMg1, 1 mm thick) were acquired from Allega GmbH (CH). Whatman qualitative filter paper grade 1 was purchased from Sigma-Aldrich (US).

### Coral-like silicone nanofilaments coating

Aluminum samples were etched via immersion in a 1 M NaOH aqueous solution for 90 s to form Al(OH)_3_ on their surface. After this, samples were immersed for 10 s in deionized water and then copiously rinsed with running deionized water. Samples were dried with a dry N_2_ stream and placed in a custom 6.6 L glass desiccator^41,42^, where they were exposed for 1 h to a N_2_ atmosphere with controlled relative humidity *RH* = (36 ± 2)% at room temperature *T* = (22 ± 1) °C. Control over the desiccator atmosphere was achieved by flushing it with a mixture of dry and water-saturated N_2_, while temperature and relative humidity were measured with an EE23 sensor (E + E Elektronik GmbH, AT) at the desiccator inlet. The desiccator was then sealed, and a mixture of TMMS (2.25 mmol) and TCMS (0.75 mmol) was injected through a septum into the desiccator. The silane mixture was let react for 2 h with the humidity present in the desiccator according to the DAGS mechanism^43^. This process produced a coating of Coral-like Silicone Nanofilaments (SNFs) on the aluminum surface, and the obtained samples are referred to as S.

### Fluorination of SNFs

S samples were activated in an O_2_ plasma chamber (Femto, Diener Electronics, DE) at 50 W for 5 min. This step introduced OH functionalities on the SNFs surface, allowing for further modification. Samples were then placed in a custom glass desiccator, as done for the S sample preparation, and exposed for 1 h to a N_2_ atmosphere with controlled relative humidity *RH* = (25 ± 3)% at room temperature *T* = (22 ± 1) °C. The desiccator was then sealed and TCPFOS (0.67 mmol) was injected and let react for 16 h. This process resulted in the modification of SNFs with fluorine-containing functionalities (f-SNFs), and the obtained samples are referred to as F.

### Fabrication of slippery surfaces

Approximately 1 µL of Silicone oil (100 cSt at 25 °C) was pipetted on the surface of S samples, which were placed vertically on grade 1 filter paper to drain excess oil for 2 days. The obtained SLIPS are referred to as LS. The same procedure was repeated with Krytox (390 cSt at 25 °C) instead of silicone oil to obtain fluorinated slippery surfaces, referred to as LF.

### Room temperature wetting analysis

Water static contact angle and sliding angle (roll-off angle) measurements were conducted on a Krüss DSA100 goniometer equipped with a PA3220 tilting device (Krüss GmbH, DE) with 10 μL of ultrapure water (MilliQ, Millipore Corporation, US). Room temperature was (23 ± 1) °C and relative humidity was (60 ± 3)%. Measurements were carried out on at least 5 different spots on each sample, and the standard deviation of the measurements was used as uncertainty. A sliding angle of 90° was used to represent complete droplet pinning on the surface. When it was not possible to firmly deposit a droplet on the surface due to excessively low contact angle hysteresis, a conservative value of 1° was used for the sliding angle.

### Sub-zero wetting analysis

A temperature control chamber equipped with a Peltier element (Krüss TC40, Krüss GmbH, DE) was installed on the Krüss DSA100 goniometer. Sample surface temperature was monitored by a Fluke 51II thermometer (Fluke Corporation, US) with a K-type thermocouple in contact with the sample surface, while air temperature at about 1 cm above the sample was measured with a Pt-100 temperature sensor (Krüss TP20, Krüss GmbH, DE). In order to prevent frosting on the sample surface upon cooling, a dry atmosphere was attained by continuously flushing the chamber with dry N_2_. The sample surface was then cooled to − 10 °C and it was let achieve thermal equilibrium for 10 min. Contact angle and sliding angle measurements were then carried out as in the room temperature wetting analysis. At least 3 different spots were analyzed for each sample.

### Optical profilometry

Profile roughness measurements were performed with an optical profilometer (contactless measuring instrument Alicona Infinite Focus G5, Alicona Imaging GmbH, AT), according to the standard ISO 4288. A 50 × magnification objective was used to image the coating areas on three different locations of the sample. The average roughness (Ra) and the mean peak to valley height (Rz) values of the roughness profile were obtained by an average of 9 measurements in different locations of the coated surface.

### Scanning electron microscopy and energy dispersive X-ray spectroscopy

Samples were cut to shape and fixed onto Al stubs with carbon glue (Plano GmbH, DE). Samples were then coated with a 10 nm thick Pt layer with a Safematic CCU-010 sputter coater (Safematic GmbH, CH) using a rotating planetary stage. High-resolution field emission SEM imaging was carried out with a Zeiss GeminiSEM 450 (Carl Zeiss AG, DE), using an electron acceleration voltage of 5 kV. Brightness and contrast of images were subjected to minor and uniform adjustments after acquisition. Additionally, Energy Dispersive X-Ray spectroscopy (EDX) and element mapping were performed using an AZTec Advanced X-MAX80 detector (Oxford Instruments, UK) with a 5 kV voltage.

### Temperature dependence of viscosity

Kinematic viscosity of silicone oil (*T*: 25 °C and 50 °C) and Krytox GPL105 (*T*: 50 °C and 70 °C) was determined with a capillary *Ubbelohde* viscometer (Capillary type II, Schott Geräte, DE) according to ISO 3105:1994. Temperature-kinematic viscosity relationships of the lubricants were calculated from the two experimental points following the ASTM D341-17 standard.

### Icing testing

Icing testing was carried out at the icing research facilities of Tampere University^49^. The laboratory containing all the equipment and samples was kept at a constant temperature of − 10 °C for the whole duration of the test. Ice was accreted on a (30 × 30) mm^2^ area of the samples surface using a customized Icing Wind Tunnel (IWiT), whose operating parameters control the type of ice obtained (rime, glaze, mixed). In the present work, mixed glaze ice was accreted for the ice adhesion test. Detailed icing wind tunnel parameters used for ice accretion can be found in previous work^36,51,68,69^, and are summarized in Table 3.

**Table 3:** Icing wind tunnel parameters for ice accretion.

Parameter	Value
Room temperature	− 10 °C
Relative humidity	(80 ± 5)%
Median volumetric diameter of droplets	31 µm (nominal)
Ice thickness	≈ 10 mm

Ice adhesion strength to the sample surface was tested with a Centrifugal Adhesion Test (CAT)^70^, where a specimen, attached to one end of a balanced glass-fiber reinforced composite beam, is spun at constant angular acceleration of 300 rpm s^−1^ with a radial spinning length *r*. Ice adhesion shear strength is calculated as in Eq. (3), where *ω* is the angular velocity at which the accreted ice block, of mass *m*_ice_, is detached from the iced area *A*:

3 $${\tau }_{\text{ice}}=\frac{F}{A}=\frac{{m}_{\text{ice}}r{\omega }^{2}}{A}$$

At least four parallel specimens were tested for each sample, and the average of their ice adhesion strengths was taken as the value for the sample. Uncertainty was quantified as one standard deviation. Ice adhesion properties for each ice accretion event was monitored using smooth Teflon tape (3 M™ PTFE Film Tape 5490, US) as a reference surface.

## Supplementary Information

Supplementary Information.

## Data Availability

The authors declare that all data supporting the findings of this study are available within the paper and its supplementary information files.
