# Supplementary material for: Coral-like silicone nanofilament coatings with extremely low ice adhesion
Source: Sci Rep. 2021 Oct 14;11:20427. doi: 10.1038/s41598-021-98215-1 (PMC8516905; doi:10.1038/s41598-021-98215-1)
Supplement: Supplementary file 1 — Supplementary Information. [file 41598_2021_98215_MOESM1_ESM.pdf]

Supplementary Information

Coral-like Silicone Nanofilament Coatings with Extremely Low Ice Adhesion

Davide Bottone[a], Valentina Donadei[b], Henna Niemelä, Heli Koivuluoto[b], Stefan Seeger[a, *]

[a] University of Zurich, Department of Chemistry, Winterthurerstrasse 190, 8057 Zurich, Switzerland
[b] Materials Science and Environmental Engineering, Faculty of Engineering and Natural Sciences, Tampere University, P.O. Box 589, FI-33014 Tampere, Finland

* Corresponding author. *E-mail address:* sseger@chem.uzh.ch (S. Seeger)

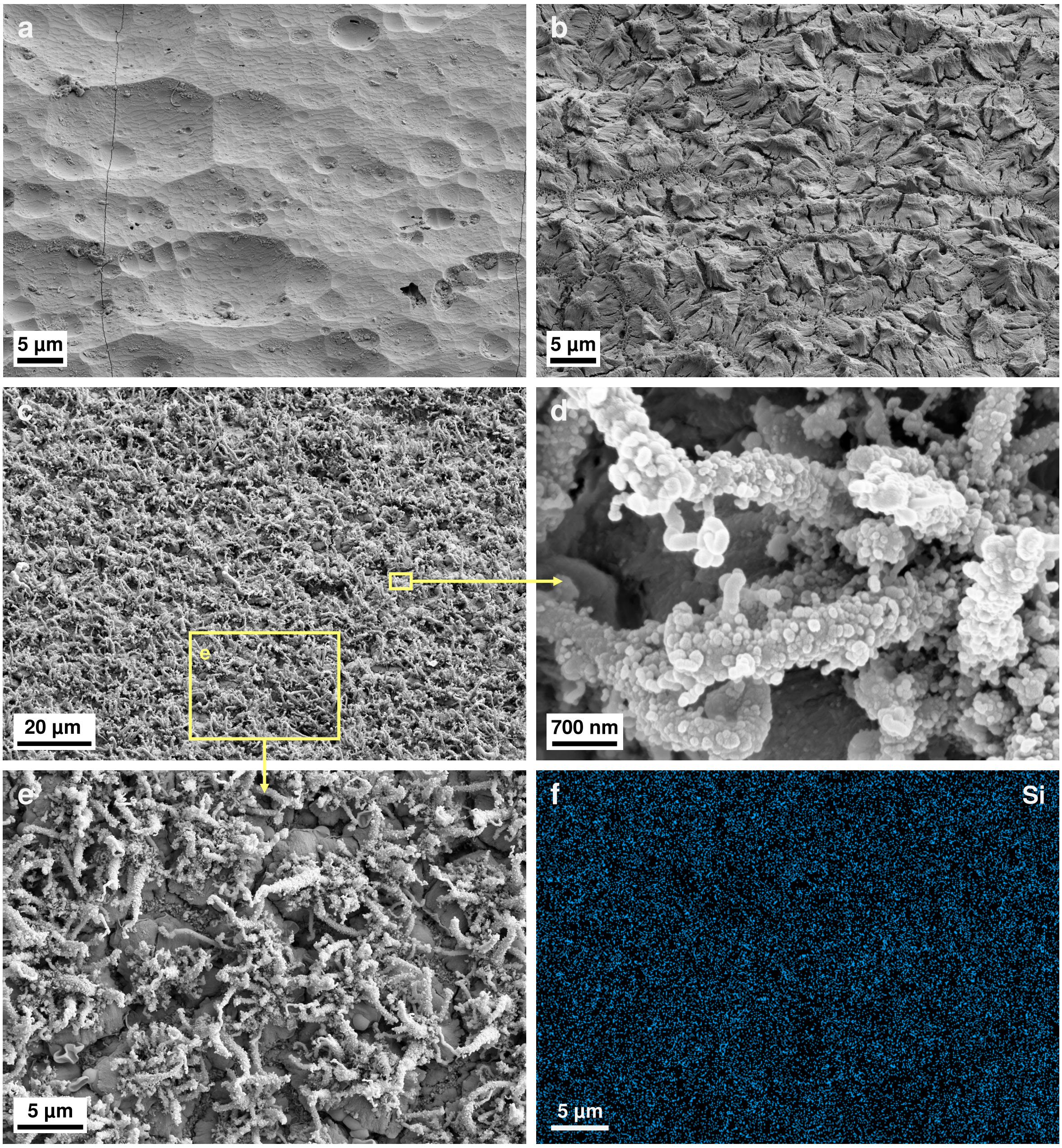

Figure S1. a-**d** SEM micrographs of: **a** bare Al substrate, **b** NaOH etched Al substrate, and **c**-**d** coral-like SNF-coated Al (sample S). **e** SEM micrograph of sample S, with **f** its Si EDX map.

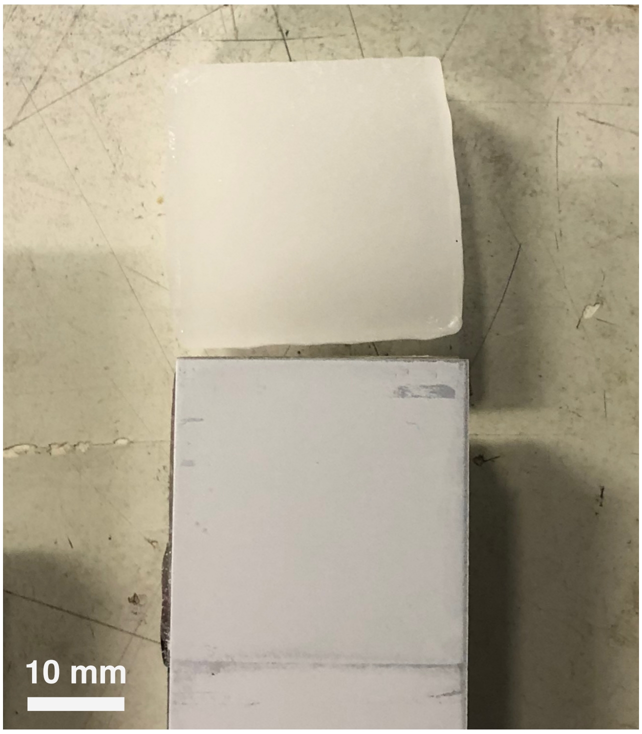

Figure S2. Ice block detached from sample F.

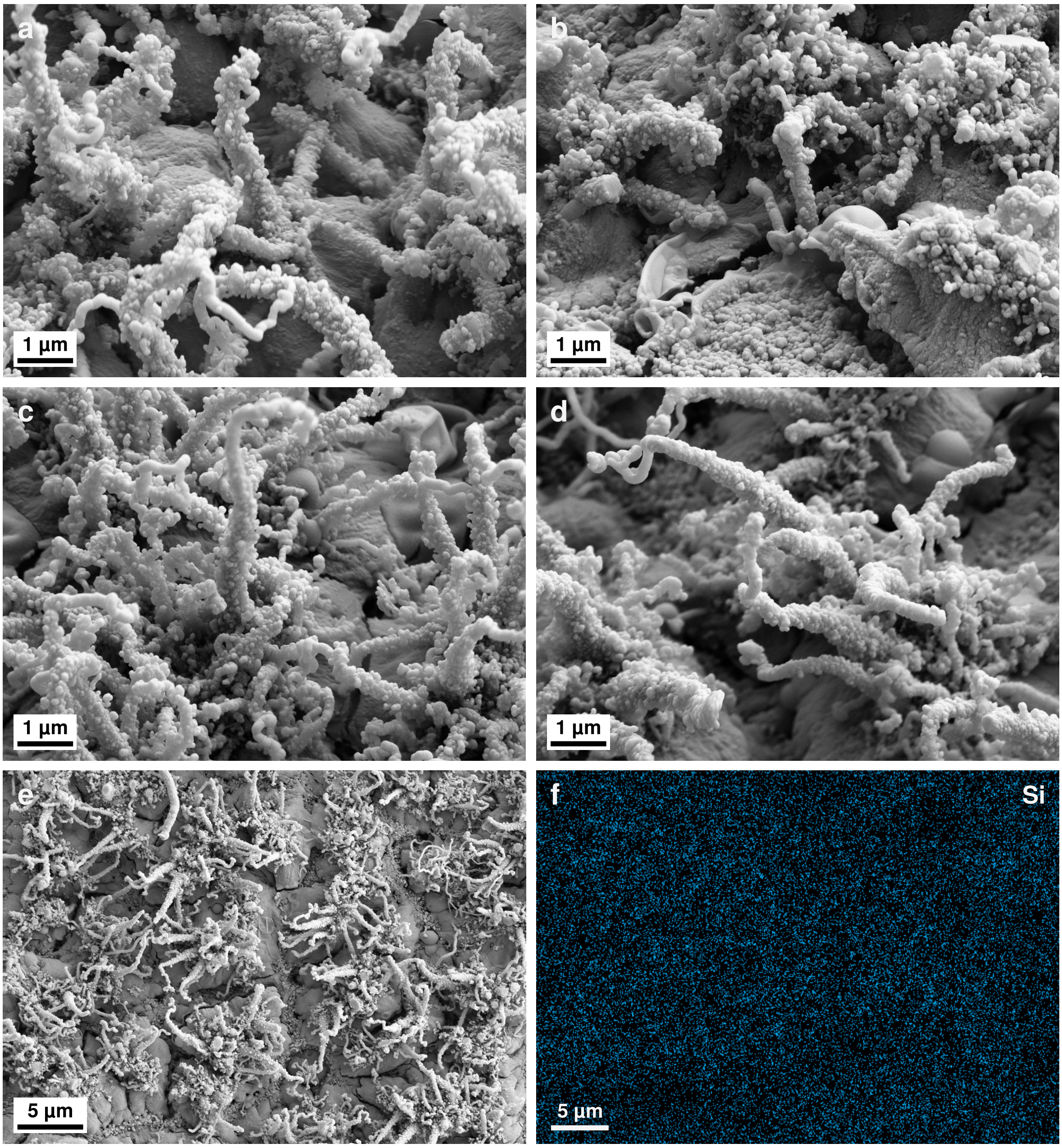

Figure S3. a-**b** SEM micrographs of: **a** pristine sample S, and **b** sample S after 5 icing cycles. **c**-**d** SEM micrographs of: **c** pristine sample F, and **d** sample F after 5 icing cycles. **e** SEM micrograph of sample F after 5 icing cycles, with **f** its Si EDX map.
